# Increased Hepatic Fatty Acids Uptake and Oxidation by LRPPRC-Driven Oxidative Phosphorylation Reduces Blood Lipid Levels

**DOI:** 10.3389/fphys.2016.00270

**Published:** 2016-07-12

**Authors:** Shi Lei, Run-zhu Sun, Di Wang, Mei-zhen Gong, Xiang-ping Su, Fei Yi, Zheng-wu Peng

**Affiliations:** ^1^College of Biological and Pharmaceutical Sciences, China Three Gorges UniversityYichang, China; ^2^Department of Psychiatry, Xijing Hospital, Fourth Military Medical UniversityXi'an, China; ^3^Department of Laboratory Medicine, Huashan Hospital North, Shanghai Medical School, Fudan UniversityShanghai, China

**Keywords:** LRPPRC, oxidative phosphorylation, fatty acids, triglyceride, mitochondria

## Abstract

Hyperlipidemia is one of the major risk factors of atherosclerosis and other cardiovascular diseases. This study aimed to investigate the impact of leucine rich pentatricopeptide repeat containing protein (LRPPRC)-driven hepatic oxidative phoshorylation on blood lipid levels. The hepatic LRPPRC level was modulated by liver-specific transgenic or adeno-associated virus 8 carried shRNA targeting Lrpprc (aav-shLrpprc). Mice were fed with a high fat diet to induce obesity. Gene expression was analyzed by quantitative real-time PCR and / or western blot. The hepatic ATP level, hepatic and serum lipids contents, and mitochondria oxidative phosphorylation (OxPhos) complex activities were measured using specific assay kits. The uptake and oxidation of fatty acid by hepatocytes were assessed using ^14^C-palmitate. LRPPRC regulated the expression of genes encoded by mitochondrial genome but not those by nuclear genome involved in mitochondria biogenesis, OxPhos, and lipid metabolism. Increased OxPhos in liver mediated by LRPPRC resulted in the increase of hepatic ATP level. Lrpprc promoted palmitate uptake and oxidation by hypatocytes. The hepatic and serum triglyceride and total cholesterol levels were inversely associated with the hepatic LRPPRC level. These data demonstrated that LRPPRC-driven hepatic OxPhos could promote fatty acids uptake and oxidation by hepatocytes and reduce both hepatic and circulating triglyceride and cholesterol levels.

## Introduction

Hyperlipidemia and hypertension are increasingly prevalent both in the United States (Go et al., 2014) and globally (Hanevold, 2013) in both adult and pediatric populations. Lipid-driven chronic arterial wall inflammation is responsible for initiation and progression of atherosclerosis which is one of the diseases causing most death (Buckley and Ramji, 2015; Zarzycka et al., 2015). Atherosclerosis is the underlying cause for myocardial infarction, stroke, incident heart failure, renal failure and other vascular diseases (Lloyd-Jones et al., 2002), which results in great life, emotional, and financial burdens.

Hyperlipidemia and non-alcoholic fatty liver diseases (NAFLD) are highly associated (Abe et al., 2012; Wang et al., 2013; Tomizawa et al., 2014). Mitochondrial dysfunction increases predisposition to intracellular lipid accumulation (Morino et al., 2006; Schrauwen et al., 2010; Su et al., 2010) and non-alcoholic steatohepatitis (NASH) (Cortez-Pinto et al., 1999; Sanyal et al., 2001; Pérez-Carreras et al., 2003; Rector et al., 2010). Mitochondrial oxidative phosphorylation (OxPhos) activities are decreased in patients with fatty liver disease (Cortez-Pinto et al., 1999; Pérez-Carreras et al., 2003). OxPhos has been manipulated to alleviate lipotoxicity and NAFLD (Lelliott et al., 2007; Pospisilik et al., 2007; Bellafante et al., 2013). Disrupting mitochondrial function by knocking out paraoxonase 3 gene (Pon3) causes significant increases of mouse plasma and hepatic cholesterol levels, hepatotoxicity markers in circulation, gallstone weight, and mortality (Shih et al., 2015). Ectopic expression of peroxisome proliferator-activated receptor γ coactivator-1β (PGC-1β) promotes mitochondrial biogenesis, lipid export, fatty acid oxidation, and OxPhos (Lelliott et al., 2007; Bellafante et al., 2013).

Leigh Syndrome French Canadian variant, a rare form of Leigh Syndrome, is caused by mutations in the gene encoding leucine-rich pentatricopeptide repeat-containing protein (LRPPRC) (Mootha et al., 2003). Although the mechanism is still controversial, LRPRPC is mainly located in mitochondria and regulates the expression of genes harbored on mitochondrial genome (Sasarman et al., 2010; Sondheimer et al., 2010; Liu et al., 2011; Ruzzenente et al., 2012; Xu et al., 2012; Harmel et al., 2013; Mourier et al., 2014). As LRPPRC has been shown to regulate mitochondrial oxidative respiration, fatty acid oxidation, and hepatic lipid content (Liu et al., 2011; Mourier et al., 2014; Akie et al., 2015), this study aims to investigate whether changes in hepatic LRPPRC-driven mitochondrial OxPhos could impact hepatic and circulating lipid levels.

## Materials and methods

### Animal models

All animal experiments were performed conform with the NIH guide for the care and use of laboratory animals and protocols were approved by the IACUC of China Three Gorges University (Yichang, China). C57BL/6 mice were purchased from the Laboratory Animal Center of Northwest University and housed in a room with controlled temperature of 22–23°C and 12 h light/12 h dark cycle. Liver specific Lrpprc transgenic mouse was established as previously described (Liu et al., 2011) and back crossed with C57BL/6 mice for at least 8 generations. C-terminal myc-tagged murine Lrpprc gene expression was under the control of transthyretin (ttr) promoter, which is liver-specifically expressed (Wu et al., 1996). The pTTR1ExV3 was a gift from Dr. Terry Van Dyke (University of North Carolina). DNA microinjection into fertilized C57BL/6 embryos was performed by the transgenic core of Northwest University. To generate liver-specific Lrpprc knockdown mice, adeno-associate virus serotype 8 shLrpprc (aav-shLrpprc) (Liu et al., 2011) and aav-shCtrl (100 μl of 10^11^ pfu/ml) (HanBio, Shanghai, China) were injected into 6-week old C57BL/6 male mice (*n* = 20 each group) through tail vein. At 8 weeks old, C57BL/6, Lrpprc transgenic mice, and aav-injected mice were randomly divided into two subgroups (*n* = 10) to be fed with either regular chow (Harlan Laboratories, Indianapolis, IN) or high-fat diet with about 55% kcal from fat (approximate fatty acid profile (% total fat): 28% saturated, 30% trans, 28% cis-monounsaturated, 14% cis-polyunsaturated) (Teklad TD-93075, Harlan Laboratories) for 9 weeks. Then the mice were fasted for 12 h before blood samples were collected and mice were sacrificed by CO_2_ euthanasia and liver tissues were flash-frozen in liquid nitrogen for further experiments.

### Histology examination

Liver histology was assessed by hematoxylin and eosin (HE) staining with standard procedure.

### Serum and liver lipid contents

Serum triglyceride, cholesterol, and free fatty acids levels were measured using Triglyceride Colorimetric Assay kit (10010303, Cayman Chemical Company, Ann Arbor, MI), Cholesterol Fluorometric Assay Kit (10007640, Cayman), and Free Fatty Acid Fluorometric Assay Kit, (700310, Cayman), according to the manufacturers' protocols. Liver total lipid was extracted with previously described method (Folch et al., 1957) and the triglyceride and cholesterol levels were measured using aforementioned kits.

### Western blot

Total tissue proteins were obtained by homogenizing mouse liver tissue in RIPA buffer (50 mM Tris-HCl pH 7.4, 150 mM NaCl, 1% Triton x-100, 1% Sodium deoxycholate, 0.1% SDS, 1 mM EDTA) including a protease inhibitor cocktail (Sigma, St. Louis, MO) and resolved on SDS-PAGE (Invitrogen, Beijing, China) before transferred to a PVDF membrane and blocked with 5% BSA in TBST (50 mM Tris, 150 mM NaCl, 0.1% Tween-20, pH 7.6) for 1 h. The membranes were incubated with primary antibody overnight at 4°C followed by washed three times with TBST then probed with appropriate secondary antibodies for 1 h at room temperature, washed as above and proteins bands were detected using ECL (EMD Millipore, Shanghai, China). Anti-LRPPRC (ab80881, Abcam, Shanghai, China), COX1 (ab14705), COX5A (ab110262), and β-Actin (ab8227) antibodies were diluted according to the manufacturer instructions.

### Quantitative real-time RNA polymerase chain reaction (RT-qPCR)

Liver total RNA was extracted using RNeasy mini kit (Qiagen, Shanghai, China). The cDNA was synthesized using SuperScript™ III First-Strand Synthesis SuperMix (Life Technologies, Shanghai, China). Quantitative PCR was done using SYBRgreen master mix (Qiagen) according to manufacturer's instruction on an ABI 7500 (Life Technologies) with following cycling program: 95°C for 2 min followed by 40 cycles of 95°C for 30 s, 58°C for 15 s, and 68°C for 30 s. The primer sequences were listed in Table 1. The relative gene expression level was calculated using 2^−Δ*ΔCt*^ method with β-actin or 18S rRNA as the internal control.

**Table 1:** **The sequences of qPCR primers used in this study**.

**Gene**	**Oligonucleotide sequences**	
ND1	CCCCTTCGACCTGACAGAAG	GGGCCGGCTGCGTATT
ND2	CAAGGGATCCCACTGCACAT	GAGTAGCGGGTAGATTTGGATTAAAA
ND4	ATCACTCCTATTCTGCCTAGCAAAC	GAAGTCCTCGGGCCATAATTATAGT
ND6	GGCCTGGAATTCAGCCTACTAG	TGTTATGTTAAGGATAAGACCGTTTGTT
CytB	AGACAACTACATACCAGCTAATCCACTAA	GAATGGCGTATGCAAATAGGAAA
COX1	TTTTCAGGCTTCACCCTAGATGA	CCTACGAATATGATGGCGAAGTG
COX3	GCAGGATTCTTCTGAGCGTTCT	GTCAGCAGCCTCCTAGATCATGT
ATP6	AATTACAGGCTTCCGACACAAAC	TGGAATTAGTGAAATTGGAGTTCCT
ATP8	GCCACAACTAGATACATCAACATGATT	GGTTGTTAGTGATTTTGGTGAAGGT
Lrpprc	TTCAGTGCTCTCGTCACAGG	GTCGCGGTCCATGAAGTAAT
PGC-1α	ATGTGTCGCCTTCTTGCTCT	ATCTACTGCCTGGGGACCTT
PGC-1β	TAGGAGGGAGGCAAACCAGA	AGTGCTTTGTGAGACTGGCA
ERRα	GCAGGGCAGTGGGAAGCTA	CCTCTTGAAGAAGGCTTTGCA
Tfam	GGGAATGTGGAGCGTGCTAA	GATAGACGAGGGGATGCGAC
Ndufs1	GCGCTTCGAGTGGTGTTTTCT	TCAAGGGCAAGAGGAGAACGG
Sdha	GCTGGTGTGGATGTCACTAAGG	CCCACCCATGTTGTAATGCA
Uqcrb	CGGGCCGATCTGCTGTT	ACCACTTTCGAAAACCATCCA
Cox5A	TGATGCCTGGGAATTGCGT	ACAACCTCCAAGATGCGAACA
Atp5S	ATTGATGCCACCGATTCTTGTA	GCTCTAGGCCCACCATGTGA
β-Actin	GGTCATCACTATTGGCAACG	ACGGATGTCAACGTCACACT
18S rRNA	AAACGGCTACCACATCCAAG	CCTCCAATGGATCCTCGTTA

### Isolation of mitochondria from mouse livers

Mitochondria were isolated from chow-fed C57BL/6, Lrpprc transgenic, aav-shControl, and aav-shLrpprc mouse livers using a previously described method (Frezza et al., 2007). Briefly, liver tissue was rinsed 4–5 time in ice-cold isolation buffer (IB, 0.01 mol/L Tris–MOPS, 0.01 mol/L EGTA/Tris, and 0.2 mol/L sucrose, pH 7.4) to clear all blood, minced into small pieces on ice, homogenized (tissue: IB = 1: 10 w/v) using a pre-cooled Teflon pestle at 1600 rpm for 4–5 strokes on ice. The homogenate was transferred into 50 ml polypropylene Falcon tube and centrifuged at 600 g for 10 min at 4°C. The supernatant was transferred into glass centrifuge tubes and centrifuged at 7000 g for 10 min at 4°C. The supernatant was discarded and the mitochondria pellet was washed twice with 5 ml cold IB and centrifuged at 7000 g for 10 min at 4°C before suspended in 1 ml IB). The mitochondrial concentration was determined by Biuret methods.

### Analysis of mitochondrial oxygen consumption

Mitochondria oxygen consumption of mouse liver was measured on a Clarke-type electrode (Hansatech Instruments, Norfolk, UK) as previously described (Liu et al., 2011) with minor modifications. The amount of mitochondria equaling 0.5 μg mitochondrial proteins was resuspended in 600 μl of respiration buffer (25 mM glucose, 1 mM pyruvate, and 1% BSA in PBS) and loaded onto the electrode. Oligomycin and myxothiazol were added sequentially to evaluate uncoupled and non-OxPhos oxygen consumption.

### Analyses of the enzymatic activities of OxPhos complexes

The enzymatic activities of OxPhos complexes and citrate synthase were measured using MitoCheck Complex Activity Assay Kits (700930 for complex I, 700940 for complex II, 700950 for complex II/III, 700990 for complex IV, and 701000 for complex V, Cayman Chemical, Ann Arbor, MI), and Citrate Synthase Activity Assay Kit (701040, Cayman Chemical) according to manufacturer's protocols.

### Measurement of liver ATP level

Liver tissue pieces (about 100 μg) were homogenized and extracted with previously described method (Ghanbari-Niaki et al., 1999). All lysates were adjusted to the same μg liver / μl before 50 μl lysate being used for measurement using a CellTiter-Glo Luminescent Cell Viability Assay kit (Promega, Madison, WI) according to manufacturer's protocol. The luminescent signal was detected using a POLARstar Omega microplate reader (BMG Labtech, Offenburg, Germany). The average liver ATP levels of Lrpprc Tg or Lrpprc knockdown mice were normalized against their respective control mice.

### Palmitate oxidation in primary hepatocytes

Palmitate oxidation was performed as previously described (Huynh et al., 2014) with modifications. Mouse primary hepatocytes were isolated from 13 to 15 week old wild type, Lrpprc transgenic, aav-shcontrol, and aav-shLrpprc male mice. Mice were anesthetized by pentobarbital sodium (30 mg/kg) intraperitoneally and hepatocytes were isolated with two-stage collagenase perfusion methods (Li et al., 2010). The hepatocytes were cultured in complete medium for 12 h followed by being incubated in starvation medium (Liu et al., 2011) for another 12 hr. Then the hepatocytes were incubated in pre-incubation medium (1% BSA, 25 mM HEPES and 0.5 mM Palmitate (P9767, Sigma-Aldrich, St Louis, MO) in DMEM) for 60 min followed by incubating with ^14^C-palmitate (0.1 μCi/uL, 59 Ci/mmol, NEC075H050UC, Perkin Elmer, Waltham, MA) for 3 h. At the end of the incubation, 100 μL of 1 mol/L percholic acid was added into each well and filter paper disk moistened with 2 mol/L KOH was immediately placed over each well. Plates were sealed and rocked gently for 60 min at room temperature. Paper disks were transferred into scintillation vials with 4 ml scintillation fluid and measured the average counts per minute over 3 min with a LS 6500 Scintillation Counter (Beckman Coulter, Beijing, China).

### Palmitate uptake by primary hepatocytes

Two procedures for palmitate uptake assay were similar to aforementioned palmitate oxidation assay with following changes.

Protocol 1. After starvation, hepatocytes were loaded with cold palmitate in pre-incubation medium for 2 h and then changed to ^14^C-palmitate for 30 min before they were washed 5 times with washing buffer (1 mmol/L EDTA and 0.01% Tween 20 in 1x PBS, pH 7.4) and counted.

Protocol 2. After starvation, hepatocytes were cultured with ^14^C-palmitate-containing medium for 1 h, washed 3 times with washing buffer, and then cultured in pre-incubation medium with cold palmitate for 90 min before being washed 5 times with washing buffer and counted.

### Statistical analysis

All data were expressed as mean ± SEM. Statistical analyses were performed using GraphPad (Prism). Differences among groups were analyzed by either Student's *t*-test or by two-way ANOVA with Bonferroni's *post hoc* test. A *p*-values less than 0.05 was considered statistically significant.

## Results

### Hepatic LRPPRC level does not influence the weight gain of mouse fed with high-fat diet

**Figure 1:**
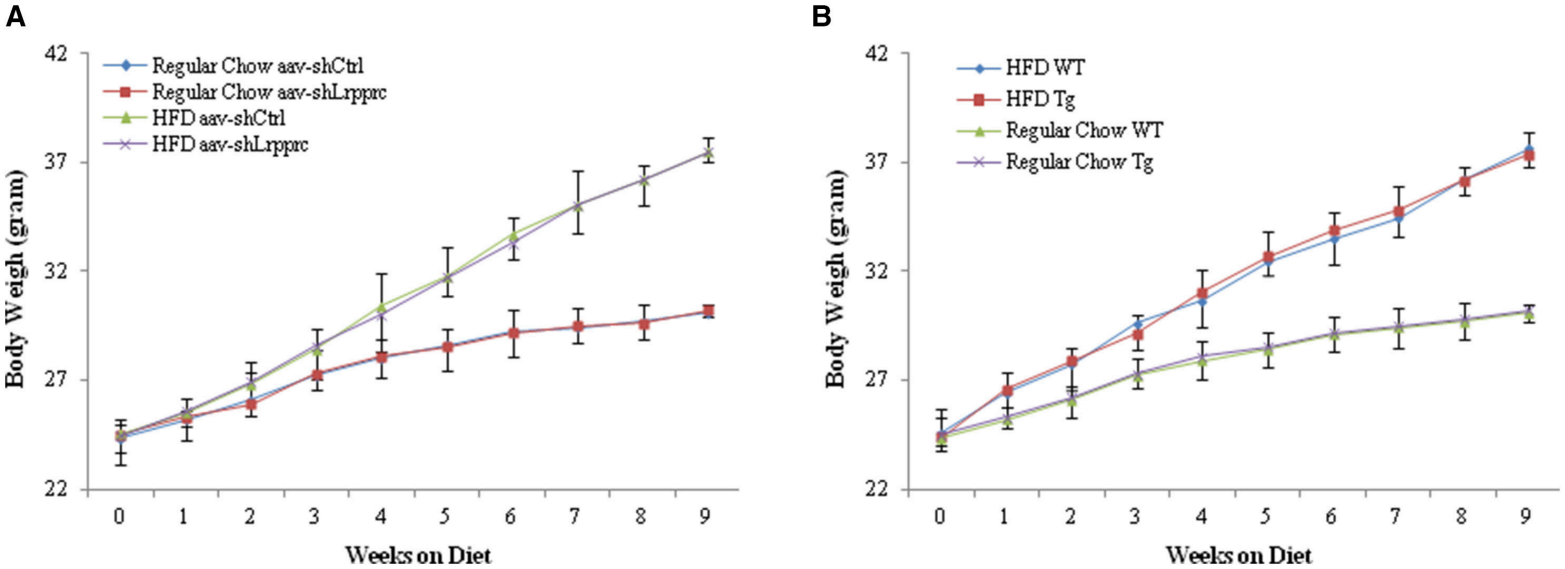
**LRPPRC does not influence mouse weight gain**. The average weight of C57BL/6 and Lrpprc liver transgenic **(A)**, aav-shCtrl and aav-shLrpprc **(B)** mice (*n* = 10) during the 9 weeks fed with regular chow or high-fat diet. Data were expressed as mean ± SEM (*n* = 10). WT, C57BL/6 mice; Tg, Lrpprc liver transgenic mice.

The initial body weight of all mice (8 week old) were 24–24.5 g. The weekly weight gains of Lrpprc transgenic mice and wild type C57BL/6 mice (Figure 1A) as well as those of aav-shLrpprc and aav-shCtrl (Figure 1B) were very similar. High fat diet caused faster weight gain in all groups (Figure 1).

### LRPPRC level regulates mitochondrial gene expression

**Figure 2:**
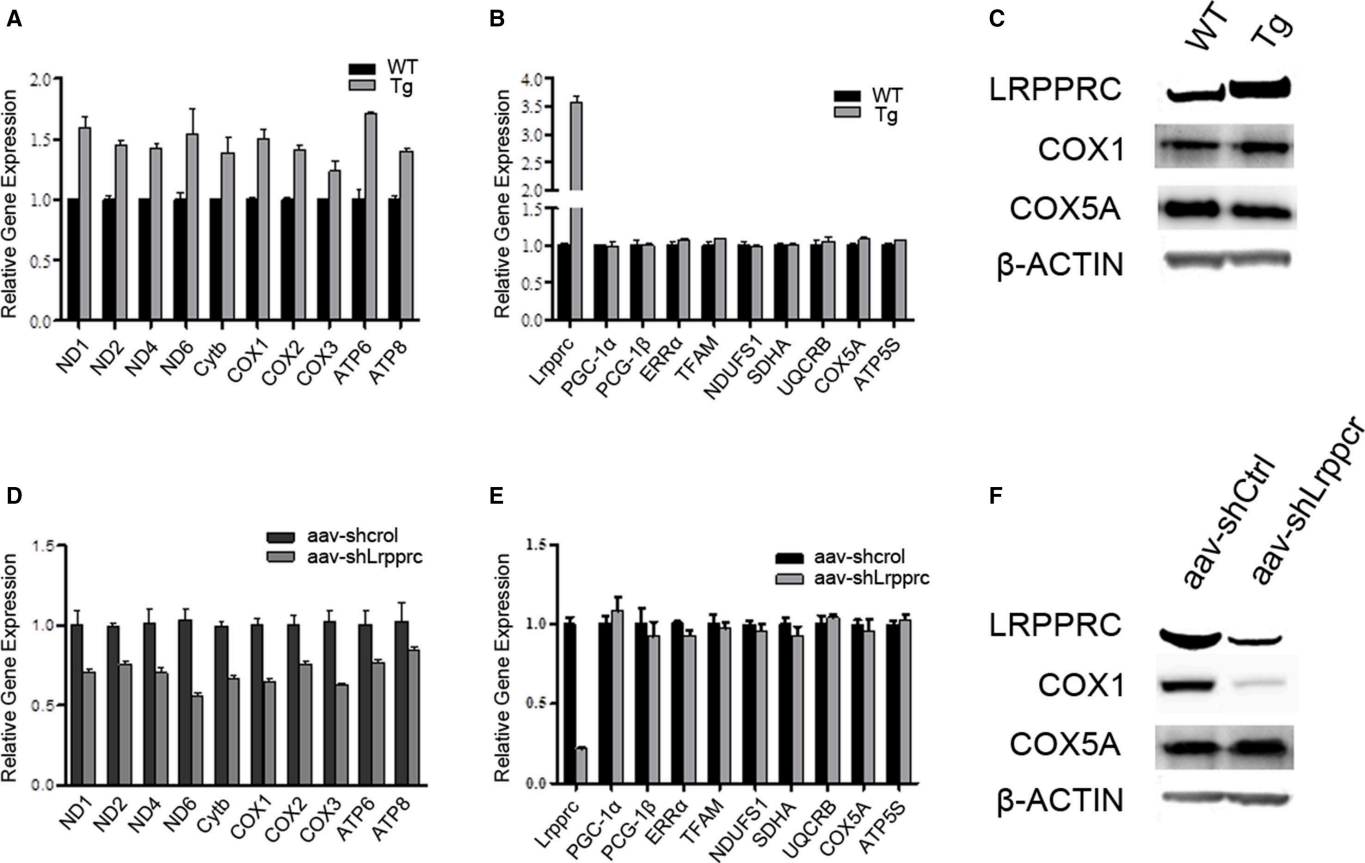
**LRPPRC upregulates the expression of mitochondrial genome encoded genes**. The relative mRNA levels of mitochondrial genome encoded genes **(A,D)** and nuclear genome encoded genes **(B,E)** of livers from WT and Lrpprc transgenic **(A,B)** or aav-shCtrl and aav-shLrpprc **(D,E)** tail-vein injected mice. The protein levels of LRPPRC, COX1, and COX5A from Lrpprc transgenic **(C)** and knockdown **(F)** mice livers. Experiments were performed in triplicates. Data were expressed as mean ± SEM (*n* = 10). WT, C57BL/6 mice; Tg, Lrpprc liver transgenic mice.

Overexpression of LRPPRC in mouse liver significantly upregulated the transcript levels of genes encoded by mitochondrial genome (mtDNA) (Figure 2A) but did not increase the mRNA levels of nuclear genes involved in either OxPhos (NDUFS1, SDHA, UQCRB, COX5A, and ATP5S) or mitochondrial biogenesis (PGC-1α, PGC-1β, ERRα, and Tfam) (Figure 2B). Accordingly, the protein level of mtDNA-encoded cytochrome c oxidase subunit I (COX1) but not that of nuclear genome (nDNA)-encoded COX5A was increased in Lrpprc transgenic mouse liver (Figure 2C). On the other hand, knockdown hepatic LRPPRC with shRNA systematically reduced the expression levels of mtDNA genes (Figures 2D,F) but did not inhibit the expression of nDNA-encoded genes involved in mitochondrial biogenesis or OxPhos (Figures 2E,F).

### LRPPRC increases hepatic ATP level and respiration rate of liver mitochondria

**Figure 3:**
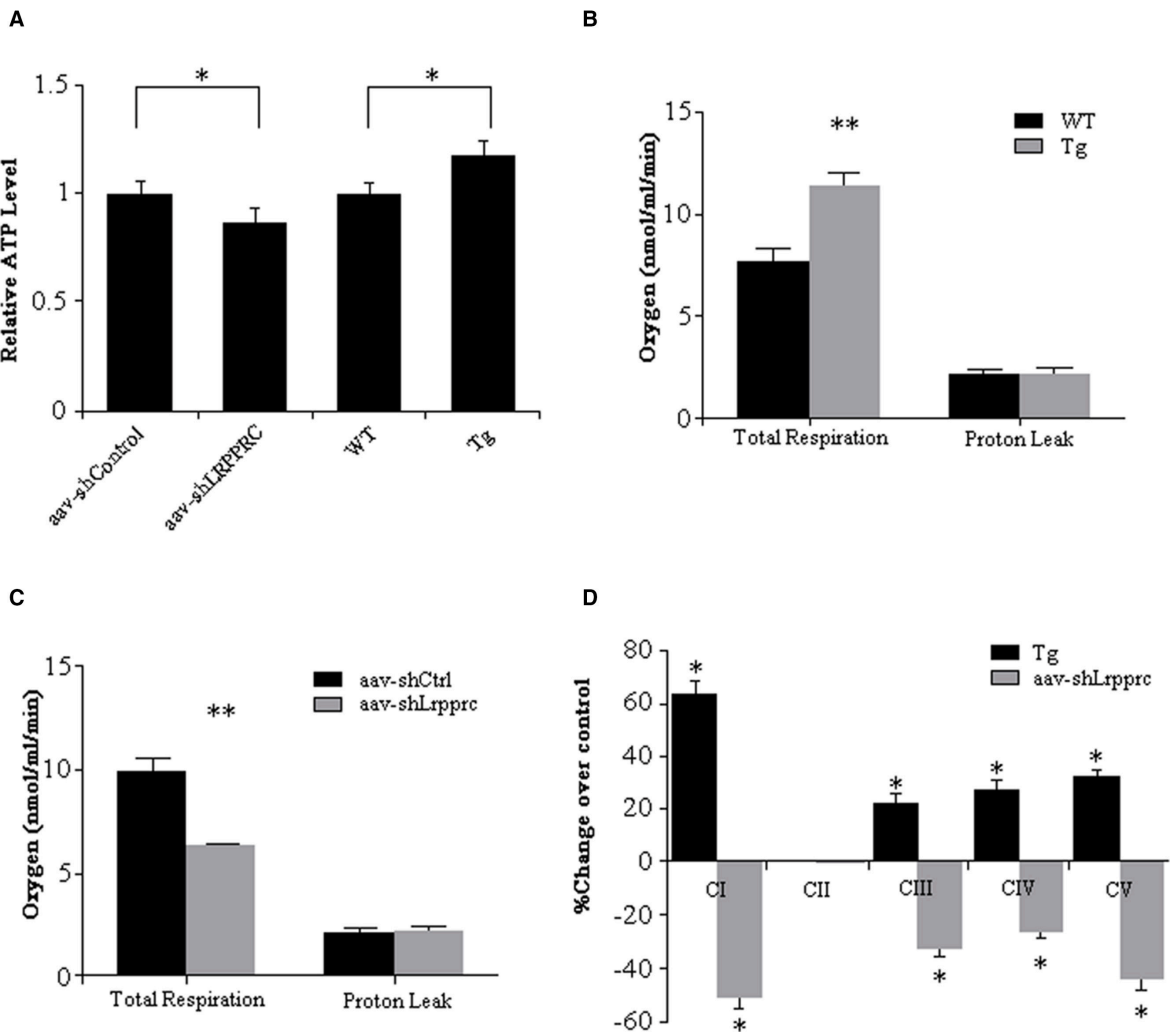
**LRPPRC increase oxidative phosphorylation and hepatic ATP level. (A)** The hepatic ATP of WT, transgenic, aav-shCtrl, and aav-shLrpprc mice was measured using CellTiter-Glo Luminescent Cell Viability Assay kit and the relative ATP levels were calculated against WT or aav-shCtrl mice. The hepatic mitochondria respiration rates of WT and transgenic mice **(B)** and aav-shCtrl and aav-shLrpprc mice **(C)** were measured using a Clarke-type electrode. Experiments were performed in triplicates. **(D)** The enzymatic activities of liver mitochondria oxidative phosphorylation complexes were assessed with specific assay kits. The results were shown as percentage change over control (transgenic compared to wild type mice and aav-shLrpprc compare to aav-shControl mice). Data were expressed as mean ± SEM (*n* = 10). WT, C57BL/6 mice; Tg, Lrpprc liver transgenic mice. ^*^p < 0.05; ^**^p < 0.01 by *t*-tests.

The hepatic ATP level of aav-shLrpprc mice was 14% less than that of aav-shCtrl mice whereas the hepatic ATP level of Lrpprc transgenic mice was increased 17% over its control (Figure 3A).

The respiration rate of mitochondria from Lrpprc liver-transgenic mouse liver was 48.8% higher than that of C57BL/6 wild type mouse liver (*p* < 0.01) with their proton leakage was essentially at the same level (Figure 3B). After knockdown hepatic LRPPRC with aav-shLrpprc, the respiration rate of mitochondria isolated from those mouse livers was 36.6% less than that of aav-shControl mouse liver mitochondria (*p* < 0.01) whereas the proton leak was at similar level between the two groups (Figure 3C).

The enzymatic activities of OxPhos complexes I, III, IV, and V were 20–60% higher in Lrpprc transgenic mouse liver mitochondria than those of wild type mice (*p* < 0.05) whereas they were significantly reduced in Lrpprc knockdown mouse liver mitochondria compared to shControl mice (Figure 3D). However, LRPPRC did not impact the activities of complex II (Figure 3D) and citrate synthase (data not shown).

### LRPPRC reduces serum triglyceride and cholesterol levels

**Figure 4:**
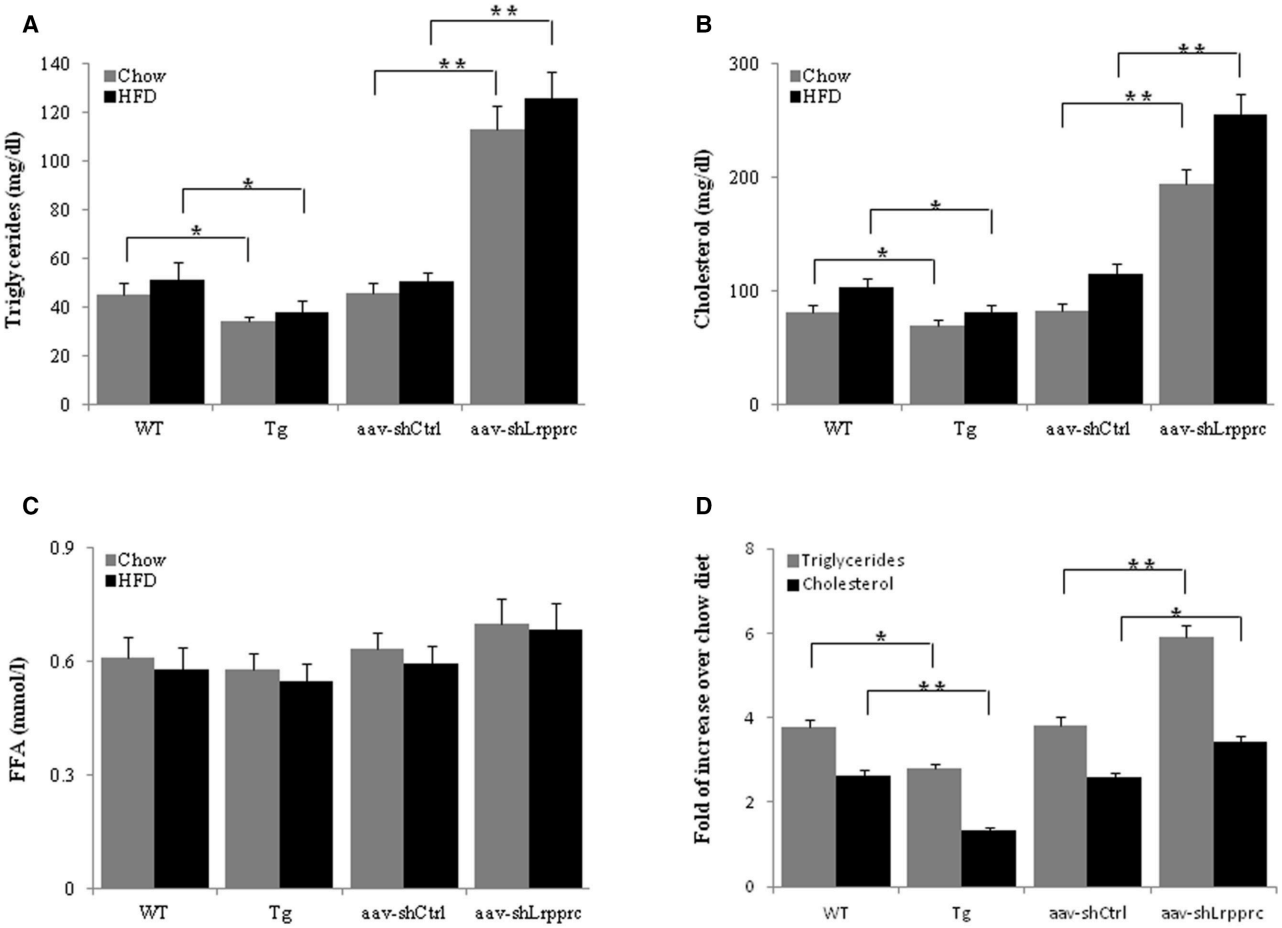
**LRPPRC reduces hepatic and serum triglyceride and cholesterol levels**. The fasting serum triglyceride **(A)**, cholesterol **(B)**, free fatty acids **(C)** levels from WT, transgenic, aav-shCtrl, and aav-shLrpprc mice after being fed with high-fat diet for 9 weeks. **(D)** The changes of hepatic triglycerides and cholesterol levels of mice fed with high-fat diet compared to regular chow fed mice. Data were expressed as mean ± SEM (*n* = 10). WT, C57BL/6 mice; Tg, Lrpprc liver transgenic mice. ^*^p < 0.05; ^**^p < 0.01 by *t*-tests.

After 9 weeks of high-fat diet feeding, the fasting serum triglyceride and cholesterol levels of Lrpprc liver transgenic mice were significantly lower than those of wild type mice (*p* < 0.05) whereas aav-shLrpprc mice had much higher serum triglyceride and cholesterol levels than shControl mice (*p* < 0.01) (Figures 4A,B). The fasting free fatty acids levels were not significantly altered by the changes of hepatic LRPPRC level (Figure 4C).

**Figure 5:**
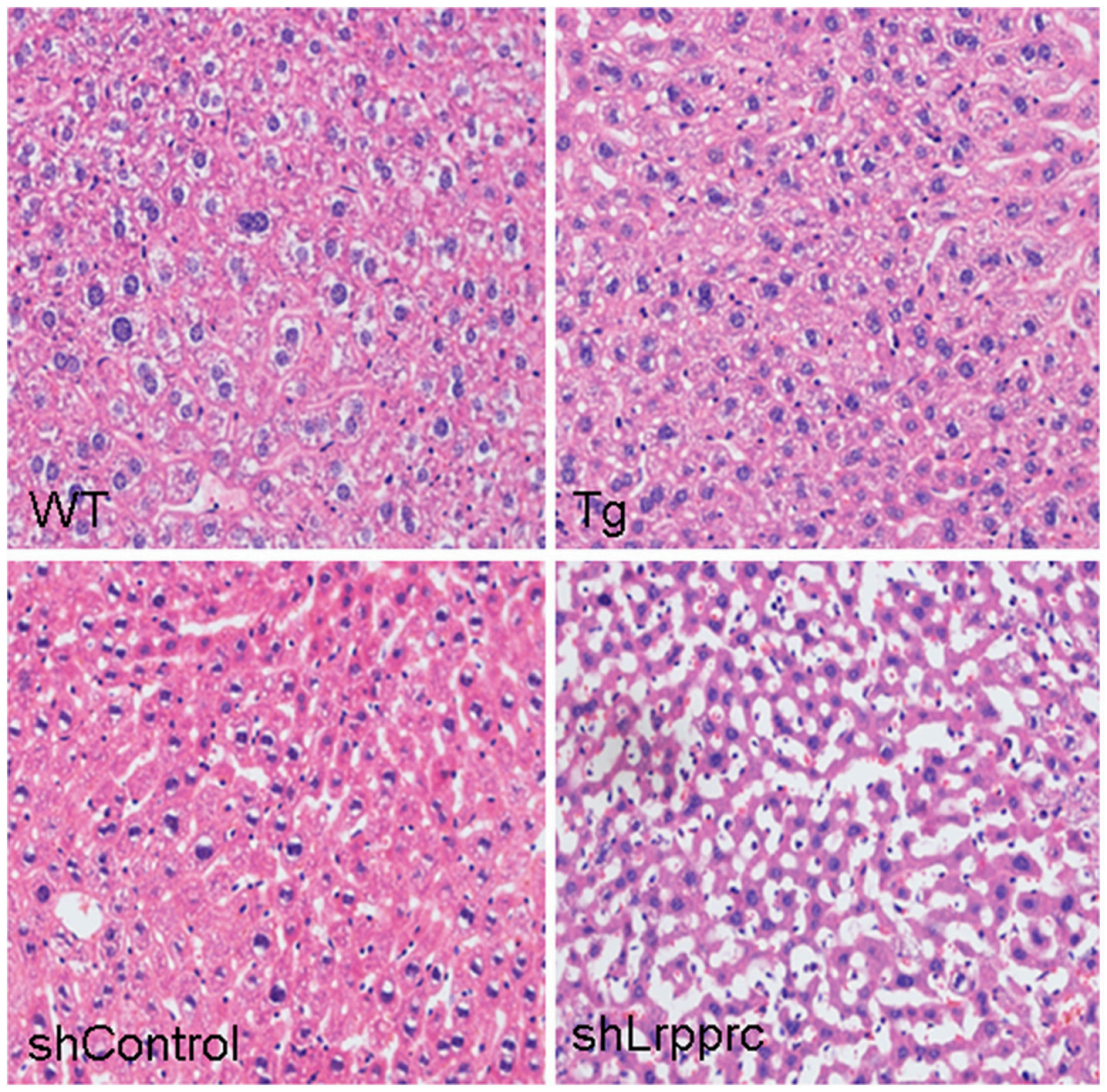
**LRPPRC protects liver from high-fat diet caused damages**. Representative images of hematoxylin and eosin staining sections of livers from wild-type mice (WT), and LRPPRC liver transgenic mice (Tg), aav-shcontrol mice, and aav-shLrpprc mice.

High-fat diet induced the accumulation of triglyceride and cholesterol in mouse liver of all genotypes studied (Figure 4D). However, Lrpprc liver-specific transgene attenuated the accumulation of hepatic triglyceride and cholesterol whereas knockdown liver Lrpprc expression augmented hepatic accumulation of triglyceride and cholesterol (Figure 4D). Meanwhile, LRPPRC protected liver from damages caused by high-fat diet (Figure 5). However, the mRNA levels of genes involved in lipid metabolism including ACSM, ACSL1, CPT-1a, MCAD, LCAD, HMGCS2, PPARα, and PPARγ were not significantly changed in the livers of either Lrpprc transgenic mice or Lrpprc knockdown mice (data not shown).

### Fatty acid uptake and oxidation in hepatocytes are increased by LRPPRC

**Figure 6:**
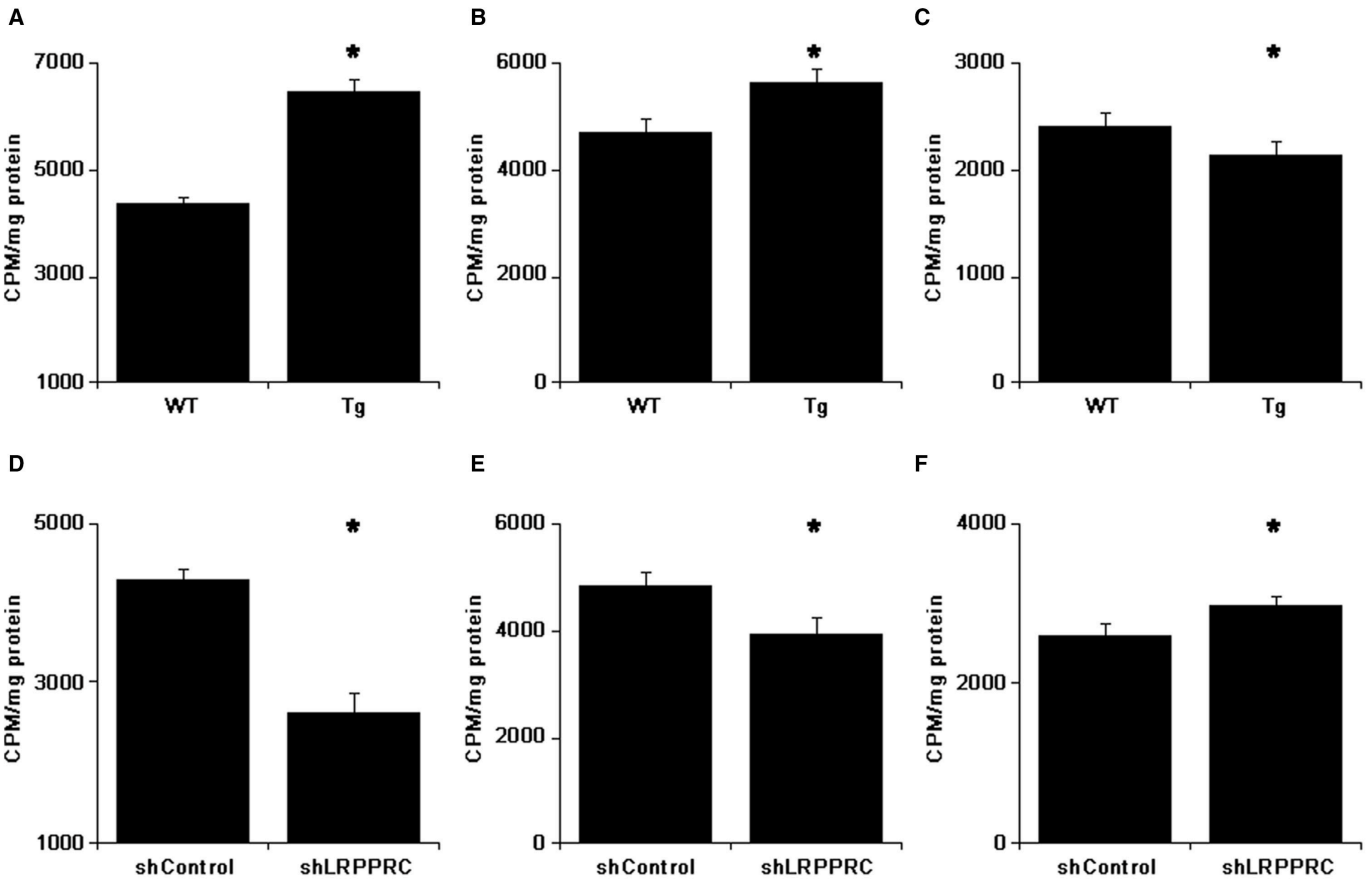
**LRPPRC promotes fatty acids uptake and oxidation by hepatocytes**. After incubated with ^14^C-palmitate for 3 h, the ^14^C in CO_2_ was counted **(A,D)**. The ^14^C counts of hepatocytes after culturing with cold palmitate for 2 h followed by incubation with ^14^C-palmitate for 30 min **(B,E)** or incubating with ^14^C-palmitate for 1 h followed by cold palmitate for 90 min **(C,F)**. The experiments were independently performed 3 times with triplicates. WT, C57BL/6 mice; Tg, Lrpprc liver transgenic mice. ^*^p < 0.01 Tg vs. WT or shLrpprc vs. shControl by *t*-test.

To further delineate the mechanisms underlying the accumulation of triglycerides and cholesterols in mouse liver and serum, primary hepatocytes isolated from mice with different Lrpprc genotypes were used to analyze palmitate uptake and oxidation *in vitro*. Hepatocytes from Lrpprc transgenic mice produced about 50% more CO_2_ than wild type hepatocytes did (Figure 6A) whereas hepatocytes from Lrpprc silencing mice produced about 40% less CO_2_ than control hepatocytes (Figure 6D).

The rate of palmitate uptake by hepatocytes with different Lrpprc levels was examined. After incubating 30 min with ^14^C-palmitate, hepatocytes from Lrpprc transgenic had about 20% more radioactive count than wild type hepatocytes (Figure 6B) whereas hepatocytes from Lrpprc knockdown mice obtained about 19% less radioactivity than control hepatocytes (Figure 6E). In a different setting, the hepatocytes were loaded with ^14^C-palmitate first and followed by chasing with cold palmitate for 2 h. The remaining radioactivity in hepatocytes from Lrpprc transgenic mice was 11% less than that in wild type hepatocytes (Figure 6C) whereas silencing Lrpprc in hepatocytes resulted in an increase of about 15% radio count (Figure 6F).

## Discussion

Overexpression of Lrpprc in mouse liver significantly elevated hepatic OxPhos through increasing the expression of OxPhos genes encoded by mitochondrial genome, which resulted in the increase of hepatic ATP levels and reduction of serum triglyceride and cholesterol levels. Moreover, Lrpprc overexpression increased the uptake and oxidation of fatty acids by hepatocytes. Knocking-down hepatic Lrpprc level caused the opposite effects. The accumulation of triglyceride and cholesterol in the livers of high fat diet fed mice was exacerbated by Lrpprc knockdown but inhibited by Lrpprc liver-specific transgene. However, Lrpprc did not have any effects on the genes involved in mitochondria biogenesis or lipid metabolism.

**Figure 7:**
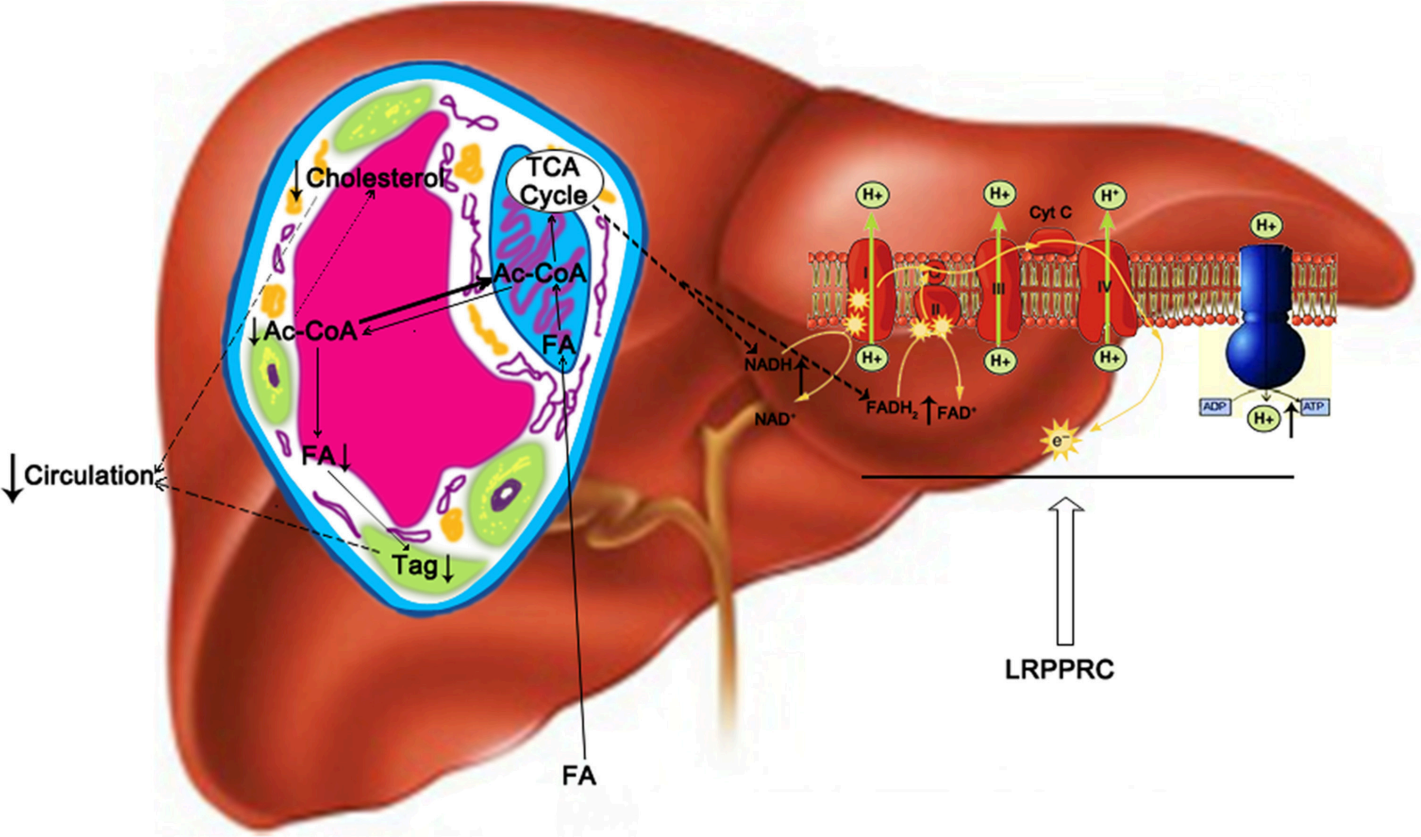
**A proposed model of how hepatic LRPPRC regulates hepatic and circulating triglycerides and cholesterol**. LRPPRC promotes the expression of genes of mitochondrial genome, resulting in more efficient oxidative phosphorylation complexes to drive fatty acid oxidation and utilization of acetyl-CoA by Kreb's cycle, which would deplete cytosolic acetyl-CoA pool and in turn slows down the *de novo* lipogenesis and cholesterol biosynthesis. Tag, triglyceride; FA, fatty acid; ac-CoA, acetyl-CoA.

Livers played a central role in lipids metabolism as it served as the major organ for fatty acid oxidation and *de novo* fatty acid biosynthesis. The imbalance between lipid influx (including lipogenesis and lipid uptake) and lipid out flux (including lipolysis and secretion into circulation) caused the changes of hepatic and serum lipid levels (Koo, 2013). It has been shown that depressed fatty acid β-oxidation due to reduced mitochondrial OxPhos protein contents resulted in elevated lipogenesis (Perfield et al., 2013) and Tg secretion (Diraison et al., 2003). Current study showed that LRPPRC significantly increased the activities of OxPhos complexes I, III, IV, and V, which had critical components encoded by mitochondrial genome. On the other hand, the activities of complex II and citrate synthase, both lacked protein encoded by mitochondrial genome, were not altered by the changes of LRPPRC level. The increased OxPhos complex activities led to enhanced fatty acid uptake and oxidation by hepatocytes as well as higher ATP level in mouse liver. LRPPRC promoted OxPhos by upregulating the expression of mitochondrial genome-encoded OxPhos genes, increasing the amount of assembled OxPhos complexes, and improving the efficiency of mitochondrial OxPhos (Liu et al., 2011), which would reduce the pool of acetyl-CoA and slow down *de novo* lipogenesis (Figure 7) without changing the expression of genes involved in lipid homeostasis. We defined this phenomenon as “OxPhos funnel effect” where efficient electron transport chain and ATP synthase complexes consumed increased amount of NADH and FADH_2_, which drove the uptake and oxidation of fatty acid and shifted the balance away from lipogenesis and releasing lipids into circulation from liver. Consequently, increased hepatic LRPPRC and OxPhos rate resulted in the reduction of serum and hepatic triglyceride and cholesterol levels (Figure 7), which would lower the risk of atherosclerosis and other cardiovascular diseases (Lamarre et al., 2013).

Liver specific PGC-1β transgene increased hepatic OxPhos and induced enzymes involved in fatty acid oxidation and hepatic VLDL secretion, which provided protection against liver lipid overload and fibrosis but adversely increased the secretion of triglyceride into circulation (Bellafante et al., 2013). In ob/ob mouse liver, the expression of genes promoting mitochondria biogenesis (PGC-1α and Tfam) was increased but mitochondrial OxPhos proteins and OxPhos activities were reduced, which caused the increase of *de novo* lipogenesis and hepatic triglyceride level (Perfield et al., 2013). LRPPRC-driven hepatic OxPhos activities promoted complete oxidation of fatty acids, reduced liver triglyceride level, and protected against NAFLD (Akie et al., 2015). The results of current study were in agreement with the reported liver protecting effects of LRPPRC (Akie et al., 2015) and further demonstrated for the first time that LRPPRC-driven OxPhos could reduce serum lipid contents via promoting hepatic fatty acids uptake and oxidation. The abundant ATP from OxPhos and acetyl-CoA from fatty acid oxidation enabled hepatocytes to produce glucose and/or ketone body for brain and other tissues to use as energy sources during fasting (Liu et al., 2011).

In conclusion, LRPPRC increases the expression of OxPhos genes encoded by mitochondrial genome and enhances OxPhos activities. Increased hepatic OxPhos capacity drives the uptake and oxidation of fatty acids by hepatoicytes, leading to the reduction of hepatic and circulating triglyceride and cholesterol levels without disturbing other cellular and physiological pathways. These data delineated a link between hepatic OxPhos level with the accumulation of hepatic and circulating lipid levels, which may yield new insight into developing strategies for preventing and treating metabolic and cardiovascular diseases.

## Author contributions

SL, RS, DW, MG, XS, FY performed the experiments. SL and ZP conceived and designed the study and wrote the manuscript. All authors read and approved the final version of the manuscript.

## Funding

This study was supported by a special grant from Chinese Ministry of Science and Technology (2013FY114000).

### Conflict of interest statement

The authors declare that the research was conducted in the absence of any commercial or financial relationships that could be construed as a potential conflict of interest.
